# Gluten Analysis and Regulation: Scientific, Analytical, and Economic Dimensions of Gluten‐Free Assurance

**DOI:** 10.1111/1541-4337.70516

**Published:** 2026-06-18

**Authors:** Bert Popping, Daniela Bartsch, Matthias Besler‐Scharf, Carmen Diaz‐Amigo, Rupert Hochegger, Barry Meikle, Mark Sykes

**Affiliations:** ^1^ FOCOS GmbH Alzenau Germany; ^2^ Chemical and Veterinary Analytical Institute Muensterland‐Emscher‐Lippe (CVUA‐MEL) Muenster Germany; ^3^ DLA ‐ Proficiency Tests GmbH Oering Germany; ^4^ Austrian Agency for Health and Food Safety (AGES), Department for Molecular Biology and Microbiology Institute for Food Safety Vienna Vienna Austria; ^5^ LGC Group Burlington Ontario Canada; ^6^ Fera Science Ltd Sand Hutton UK

## Abstract

Gluten‐free regulations require analytical systems that can reliably measure gluten in wheat, rye, and barley, and, in some jurisdictions, in oats across diverse food ingredients and processed foods. Examples include baked goods, fermented products such as beer, and extruded snack foods, where processing can alter gluten extractability and antibody recognition. This review examines the scientific, analytical, and regulatory factors that determine whether gluten quantification can support robust enforcement of the 20 mg gluten/kg threshold used in most jurisdictions, with a focus on method performance criteria (MPCs). The molecular complexity of gluten and the structural changes induced by baking, fermentation, and other processing steps influence extractability and epitope availability. These effects contribute to variable results across enzyme‐linked immunosorbent assays that use different antibodies, extraction chemistries, and calibration standards. The widespread use of PWG gliadin (gliadin reference material) as a calibrant supports harmonized calibration for wheat‐based analyses. Still, it can introduce bias because it does not reflect the gluten composition in processed or mixed‐cereal foods. Improved incurred reference materials offer advantages but are not yet widely accessible. Proficiency testing data from DLA and FAPAS show that interlaboratory agreement is strongest for unprocessed matrices and decreases in baked, fat‐rich, or fermented foods. These findings highlight the need for harmonized validation and for statistical criteria that define acceptable assay performance around the regulatory threshold. Advanced techniques such as liquid chromatography–tandem mass spectrometry provide detailed peptide‐level information and can support confirmation in matrices where immunoassays lose sensitivity. A coordinated, performance‐based framework aligned with Codex guidance would enable analytical methods to be evaluated against common criteria for recovery, precision, and detection capability. Codex alignment also introduces method dependency, as the current Type I Codex approach for gluten relies on the R5 Méndez ELISA as the defining method. Because Type I methods are empirical defining methods, results are comparable to the definition established by that method rather than necessarily being traceable to an independent reference value for gluten. The use of a single Type I method can therefore limit comparability when laboratories use assays based on other antibodies or extraction and calibration systems, particularly in processed matrices. Integrating such MPCs into regulatory practice, proficiency testing, and private certification systems would improve the comparability of gluten testing results and improve the reliability of gluten‐free labeling in international markets.

## 1 Introduction

Demand for gluten‐free foods has expanded markedly over the past two decades, moving from a medical dietary requirement to a global market segment with sustained commercial growth. Beyond coeliac disease, individuals with non‐coeliac gluten sensitivity or wheat allergy—each involving distinct pathophysiological mechanisms—as well as those following a gluten‐free diet for personal or lifestyle reasons, rely on accurate gluten labeling to guide food choices. Market analyses estimate the value of the gluten‐free products market at billions of euros (Grand View Research, n.d.). This growth has increased the need for analytical systems that can verify gluten‐free status with sufficient precision to support consumer protection, regulatory enforcement, and equitable trade.

In most areas of the world, the prevalence of coeliac disease is approximately 1%–2% in the general population (Gatti et al. 2024). It is triggered by immunogenic gluten peptides that resist digestion and induce an autoimmune response. Even low exposure levels can cause intestinal damage, nutrient malabsorption, and long‐term complications such as osteoporosis (Stazi et al. 2008), infertility, and lymphoma (Laurikka et al. 2022). Individuals with wheat allergy, non‐coeliac gluten sensitivity, and coeliac disease, all disorders having distinct underlying mechanisms, manage their conditions through strict avoidance of gluten‐containing cereals. The latter are defined in Annex II of EU regulation 1169/2011/EC (European Union 2025) as wheat, rye, barley, oats, spelt, kamut or their hybridized strains, and products thereof. For all affected groups, accurate gluten quantification is necessary to ensure that labeling conveys reliable information on product safety.

International regulation is largely based on the Codex Alimentarius Standard CXS 118–1979, which defines foods containing less than 20 mg gluten/kg as gluten‐free. This threshold, adopted following clinical challenge studies and risk assessments (Catassi et al. 2007), serves as the basis for legislation in many jurisdictions, including the European Union, the United States, and Canada. Compliance and enforcement activities depend on analytical methods capable of detecting gluten at concentrations close to or below this limit. Under Regulation (EU) No 1169/2011, cereals containing gluten must be declared when used as ingredients. Analytical testing is therefore primarily used to verify gluten‐free claims and detect unintended contamination from cross‐contact during production. Analytical results quantify gluten but do not distinguish whether it originates from a declared ingredient or from unintended cross‐contact. Interpretation, therefore, depends on the ingredient list and on whether the product carries a gluten‐free claim. When a test incorrectly classifies a product as exceeding 20 mg gluten/kg, manufacturers may face product withdrawal and economic losses. Conversely, underestimation poses direct health risks and can lead to regulatory action or liability.

The molecular diversity of gluten makes analytical accuracy difficult (C. Diaz‐Amigo and Popping 2012; C. Diaz‐Amigo and Popping 2013). Gluten is a collective term for a complex group of over 100 proteins present in wheat, rye, barley, and related grains (P. Shewry 2023). Depending on their solubility, they are further categorized into two protein fractions, prolamins and glutelins. These fractions adopt different names depending on the cereal source. For example, prolamins are named gliadins in wheat, hordeins in barley, and secalins in rye. Glutelins from wheat are called glutenins. The gluten composition varies depending on cereal species and cultivar, and it is responsible for the quality and unique viscoelastic properties of gluten‐containing products (Delcour et al. 2012). Processing steps such as baking, fermentation, and enzymatic hydrolysis can modify the physicochemical properties of gluten. All these factors, including gluten composition and processing effects, impact the efficiency of methods for extracting gluten from food matrices and the ability of analytical methodologies to detect gluten proteins accurately (C. Diaz‐Amigo and Popping 2012; C. Diaz‐Amigo and Popping 2013).

Most routine testing relies on enzyme‐linked immunosorbent assays (ELISAs), which differ in extraction chemistry, antibody specificity, and calibration standards. Sandwich ELISAs detect mostly intact proteins and are suited to analyze minimally processed foods (e.g., milled cereal flours, dry baking mixes, and powdered infant foods, without fermentation or high‐temperature heat treatment), while competitive ELISAs are more suitable for the analysis of peptides in fermented or hydrolyzed matrices (C. Diaz‐Amigo and Popping 2012; C. Diaz‐Amigo and Popping 2013). Other methods, such as liquid chromatography–tandem mass spectrometry (LC–MS/MS) and polymerase chain reaction (PCR), are used for confirmatory analysis or species verification. PCR does not reliably quantify gluten proteins because DNA content does not consistently correlate with protein concentration across matrices. LC–MS/MS can confirm the presence of specific gluten peptides but is less widely used in routine testing owing to its complexity and resource requirements.

The absence of harmonized reference materials is a persistent limitation across all method types. Many ELISA kits are calibrated against the Prolamin Working Group (PWG) gliadin (Bugyi et al. 2012; Van Eckert et al. 2006), which does not represent the full gluten profile of cereal flours or processed foods. Other material candidates have been investigated, including incurred materials that are not available or not widely adopted.

Analytical uncertainty has regulatory and practical consequences. A systematic review (Guennouni et al. 2022) found that approximately 4.66% of meals labeled gluten‐free in food service settings exceeded the 20 mg gluten/kg threshold, demonstrating that both process controls and analytical methods influence real‐world outcomes. This is particularly relevant in food service, where varied recipes and shared preparation environments increase cross‐contact risk and reduce the usefulness of occasional end product testing compared with preventive allergen management. Certification schemes and third‐party audits can mitigate these risks, but their effectiveness depends on the quality and comparability of the analytical methods applied.

This review examines the scientific basis of gluten detection, the performance of analytical methods across food categories, and the regulatory structures that depend on them. It evaluates the factors that influence analytical accuracy and discusses how harmonized performance criteria could support more consistent enforcement of gluten‐free regulations.

## 2 Gluten Protein Variability and Process‐Induced Modifications

The design and development of analytical methodologies to accurately quantify gluten start by understanding the complexities of gluten, from the molecular composition to how gluten proteins interact among themselves and with the food matrix components during processing. These factors are important for designing and validating gluten analytical methods because they can affect the gluten structure, digestibility, solubility, peptide integrity, and immunogenicity.

### 2.1 Gluten Composition and Species‐Dependent Variability

The proportions and molecular structures of gluten proteins, and the ratios between prolamins and glutelins, differ among cereal species and cultivars, influencing technological functionality and analytical responses (Barak et al. 2015; Wieser 2007).

Wheat is the most widely studied source of gluten and serves as the reference point for both functional and analytical research, because it is a major global staple crop and its gluten protein fraction has unique properties that allow the production of bread, baked goods, noodles, and pasta (P. R. Shewry and Hey 2015). Wheat gliadins fall into α‐, γ‐, and ω‐subclasses, each with distinct electrophoretic mobility and amino acid profiles. Their high content of proline and glutamine residues confers resistance to digestion and enables the formation of repetitive sequences that trigger immune responses in coeliac disease (Daly et al. 2020; Tian et al. 2015; Wieser et al. 2023). Glutenins, by contrast, consist of high‐ and low‐molecular‐weight subunits that form disulfide‐linked polymers (P. Shewry 2023) and are responsible for dough extensibility. The HMW subunits provide the structural framework, while LMW subunits modulate polymer length and influence elasticity (Edwards et al. 2003; Wieser 2007; Wieser et al. 2023). Environmental conditions, including nitrogen application and harvest maturity, also affect the gliadin‐to‐glutenin ratio (Wieser 2007).

Rye and barley contain homologous prolamin proteins, secalins and hordeins, that differ structurally and immunologically from wheat gliadin. Rye prolamins include ω‐ and γ‐secalins, with molecular weights that partially overlap with wheat gliadins. Barley hordeins are grouped into B‐, C‐, D‐, and γ‐classes, with D‐hordeins structurally resembling HMW glutenins but differing in amino acid repeat motifs and folding behavior (Schalk et al. 2017).

The ratio of gliadins to glutenins differs among wheat varieties. This compositional balance affects technological performance: soft wheat types with higher gliadin content are preferred for biscuits and cakes, while bread wheat relies on a higher glutenin fraction for dough strength (Gasparre and Rosell 2023; Khatkar et al. 2002). These differences also influence gluten quantification. Wheat genotypes express α‐ and γ‐gliadins in varying amounts, and their epitope profiles can lead to variable assay responses even at equivalent gluten concentrations.

Recent proteomic studies confirm this variability. Daly et al. (2020) used in silico epitope mapping and LC–MS/MS to compare gluten immunogenicity across cereals. They found the highest abundance of coeliac‐toxic peptides in wheat, followed by barley and rye. At the same time, oats showed much lower levels, consistent with their differentiated regulatory treatment in some jurisdictions (European Union 2014). Such differences in gluten composition across cereals highlight an analytical limitation when immunoassays use wheat‐based calibrants and antibodies targeting specific prolamin targets, because antibody reactivity differs between wheat, rye, and barley proteins (Lexhaller et al. 2016; Scherf and Poms 2016; Xhaferaj et al. 2023). For example, antibodies used in gluten immunoassays targeting R5 and G12 were developed against rye (Kahlenberg et al. 2006) and wheat peptides, respectively (Morón et al. 2008), and show differing sensitivities to wheat, rye, or barley proteins (Pahlavan et al. 2016). This can lead to over‐ or underestimation when quantifying gluten from different cereal sources (Lexhaller et al. 2016). In addition, several ELISA kits are calibrated against the PWG gliadin, a wheat‐derived prolamin standard that does not represent the protein composition of rye or barley. When reference materials do not reflect the target proteins, assay results become less comparable across cereals. To ensure accurate detection, assay validation should include rye‐ and barley‐based products, including processed materials. For accurate quantification, detection systems must be adapted to the grain source and, potentially, even to the variety (Schopf and Scherf 2018).

### 2.2 Effects of Food Processing on Gluten Structure and Detection

Food processing, including physical, chemical, and enzymatic treatments, can alter gluten proteins by affecting their molecular structure, solubility, and epitope accessibility (Magalhães et al. 2025). Such alterations affect both the functional properties of gluten in foods and its recovery during analysis.

During dough preparation, the initial formation of the gluten network occurs upon hydration of wheat flour, where gliadins (contributing viscosity and extensibility) and glutenins (contributing strength and elasticity) interact via covalent disulfide bonds and noncovalent bonds (hydrogen, ionic, and hydrophobic). Cysteine residues, enriched in α‐ and γ‐gliadins, play a key role by forming inter‐ and intramolecular disulfide bonds that determine polymer length and stability (Wang et al. 2015). Hydrogen bonding and hydrophobic interactions further stabilize the gluten matrix, enabling it to stretch and retain gas during baking. Specific glutenin alleles contribute to these functional differences. For example, 1Dx5 and 1Bx7 alleles are associated with stronger, more elastic gluten structures, whereas others, such as 1Dx2, produce weaker polymeric assemblies (P. Shewry 2023).

Heat exposure changes gluten protein structure. Processes such as baking, roasting, extrusion, and autoclaving can denature proteins, cause aggregation and cross‐linking, and reshuffle disulfide bonds (He et al. 2025). Maillard reactions between amino groups and reducing sugars introduce additional covalent linkages, making proteins more resistant to solubilization (Magalhães et al. 2025; Schirmer and Scherf 2023; Wagner et al. 2011). These effects can develop during baking, drying, or storage and often coincide with changes in food color and flavor. Processing temperature, moisture levels, and matrix pH influence the extent of these changes. Baking reduces gluten extractability to 60%–80% of that in raw dough, with the lowest recoveries observed in pretzel crust after alkaline treatment (Schirmer and Scherf 2023). Alkaline treatments can form strong protein cross‐links that may remain insoluble even after reduction‐based extraction. Further evidence is needed to quantify their impact. The food matrix also plays a role. Sugar and fat can stabilize proteins against thermal degradation or impede solvent access during extraction. In high‐sugar matrices, such as confectionery fillings, recovery rates can fall below 30% when analyzed by immunoassay. In contrast, starch‐ or meat‐based matrices generally show much higher recovery (Food Standards Agency 2023). These differences show that the impact of heating on gluten recovery depends strongly on the composition of the food matrix.

Other physical treatments, such as high‐pressure processing and extrusion, affect gluten structure by disrupting hydrogen bonding and promoting protein aggregation. The altered proteins may become less soluble yet still retain epitopes relevant to coeliac disease. These structural changes are largely irreversible and can reduce the proportion of gluten that remains extractable, leading to lower measured concentrations in finished products than in the original raw materials.

Gluten hydrolysis results in the modification of gluten proteins, the formation of peptides, and deamidation (Gabler and Scherf 2020). There are two types of hydrolytic processes. Enzymes, including proteinases and peptidases, mediate enzymatic hydrolysis, as is typical of fermented products such as beer (Pourmohammadi and Abedi 2021). The second type of hydrolysis occurs through chemical treatment with acid or alkali (Pourmohammadi and Abedi 2021). In general, hydrolysis increases gluten solubility, can improve technological properties (e.g., emulsifying and binding capacity), and can expose epitopes that are otherwise hidden within gluten protein structures (Kroghsbo et al. 2014; Pourmohammadi and Abedi 2021).

The type and extent of enzymatic or microbial fermentation influence peptide profiles. While extensive gluten hydrolysis can destroy toxic gluten peptides, partial hydrolysis can reduce peptide size; some may remain sufficiently large to retain their immunogenic activity and toxic capacity. Extended fermentation using targeted enzymes may reduce the coeliac‐toxic α‐gliadin 33‐mer, whereas shorter processes often fail to eliminate key sequences (Panda and Garber 2019).

In hydrolyzed or fermented products such as beer and soy sauce, gluten is broken into fragments that may no longer contain two binding sites, making them undetectable by sandwich ELISAs (Lacorn et al. 2018). For example, enzyme‐treated beers and malt extracts have tested below 20 mg gluten/kg by ELISA while still containing coeliac‐toxic peptides detectable by LC–MS/MS (Panda and Garber 2019; Röckendorf et al. 2017). These findings show that a negative test result does not necessarily indicate biological safety. Competitive ELISA formats can detect these smaller fragments. However, their performance depends on the extraction conditions and the integrity of the epitope. In addition, deamidation can reduce antibody binding efficiency, particularly of R5 and G12 (Kanerva et al. 2011), potentially leading to an underestimation of gluten content. The immunological impact of deamidation depends on the site and extent of modification and cannot be predicted solely from peptide size. These factors make gluten quantification in fermented foods particularly difficult.

While sandwich ELISAs are often ineffective for small peptides that lack multiple epitopes, mass spectrometry is less limited in this respect. Once suitable target peptides have been identified, LC–MS/MS can detect coeliac disease‐active peptides in partially hydrolyzed and fermented food products, including fragments that persist after extensive protein modification (Tissen et al. 2026). Method selection should consider the processing and composition of each food matrix, because these factors affect which gluten proteins or peptides remain detectable.

## 3 Analytical Method Validation Framework

Laboratories need to measure gluten accurately to support the enforcement of gluten‐free labeling, for example, Regulation (EU) No 828/2014 (European Union 2014) in Europe. While testing technology has advanced, between‐laboratory and between‐matrix variability remain challenges. Differences in antibody specificity, extraction techniques, calibration materials, and validation design continue to influence results. Consistency of results depends on thorough method validation and the use of well‐defined performance criteria.

### 3.1 Overview of Analytical Approaches

#### 3.1.1 Antibody‐Based Assays

ELISAs remain the principal tool for gluten determination in food testing laboratories, which detect gluten using poly‐ or monoclonal antibodies directed against gluten proteins or peptide sequences, often derived from prolamin fractions. ELISAs are widely adopted due to their sensitivity, ease of use, and compatibility with routine laboratory workflows. Two formats are commonly used: sandwich ELISAs detect intact gluten proteins and are suitable for unprocessed or minimally processed foods, while competitive ELISAs are designed for hydrolyzed or fermented samples where proteins are fragmented (Panda and Garber 2019). Other analytical techniques complement ELISA by extending detection, especially for gluten in more complex or processed matrices. LC–MS/MS is increasingly used for method validation and confirmatory testing because it offers high specificity and enables direct analysis and identification of individual gluten peptides. PCR‐based assays are also used for species verification when confirmation of cereal origin is required. These additional methods strengthen analytical reliability across different food types and support regulatory enforcement.

The R5 monoclonal antibody (Méndez et al. 2005) is used in the current Codex Type I ELISA procedure for gluten determination; R5 recognizes the QQPFP motif and related motifs (e.g., QQQFP, LQPFP, QLPFP) that occur in prolamins from wheat, rye, and barley, but quantitative response differs by grain type and processing history (Hochegger et al. 2015; Kahlenberg et al. 2006; Lacorn et al. 2019). It serves as the basis for several ELISA kits. Although this broad cross‐reactivity is useful for screening gluten‐containing cereals, response intensity varies with grain type and processing history. The G12 monoclonal antibody recognizes an epitope within the α‐gliadin 33‐mer and binds most strongly to wheat peptides. Its weaker response to rye and barley can limit cross‐species comparability (Garcia‐Calvo et al. 2024). Other antibodies have also been developed to target different peptide motifs. The Skerritt monoclonal antibody, also referred to as 401.21 in some commercial kit documentation, recognizes glutenins as well as gliadins, which alters its response profile compared with gliadin‐focused antibodies. However, Skerritt ELISAs (using ethanolic extraction) showed reduced detection of gluten from heat treatment or in complex matrices (Yu et al. 2021) and barley (C. Diaz‐Amigo and Popping 2012). More recent antibodies include 2D4, which was raised against a variably deamidated form of an R5‐related epitope and has been reported to improve detection in matrices where deamidation or strong processing affects immunoreactivity. In addition to gliadin‐focused assays, some kits aim to cover a broader range of gluten proteins, such as the RIDASCREEN Total Gluten ELISA, which has undergone collaborative study and is available as an option when the analytical question requires coverage beyond prolamin fractions. Because each antibody detects different peptide motifs, results from separate ELISA kits cannot be directly compared without cross‐validation. Comparative studies report over 50% variation in measured gluten concentrations when different kits are applied to the same sample, particularly in processed foods where epitope accessibility is altered (Amnuaycheewa et al. 2022; Fernández‐Gil et al. 2021). This variation demonstrates the need for clearly defined performance benchmarks specifying minimum recovery, repeatability, and allowable bias across antibody systems.

A challenging aspect of gluten quantification in assays specific to gluten fractions (e.g., gliadins) is the need to apply conversion factors to convert gliadin measurements into a gluten concentration. The factor applied is typically 2, which assumes that prolamins account for 50% of the total gluten content, with glutelins accounting for the remaining 50% (Codex Alimentarius 1979). Despite this value being applied universally, it is not free of controversy (C. Diaz‐Amigo and Popping 2013). Research has shown that the ratio of prolamins to glutelins varies across cereals and cultivars, potentially over‐ or underestimating the true gluten content in a food sample. In addition, the gliadin‐specific ELISA detection and conversion factors are not useful in products such as wheat starch, which is enriched in glutenins (Scherf 2016). Gluten content is underestimated because these methods do not detect the glutenin fraction, and applying a factor of 2 is not appropriate in this case.

Beyond laboratory‐based ELISAs, antibody systems are also applied in rapid test formats such as lateral flow devices (LFDs). These rapid immunoassay formats are widely used in food manufacturing for on‐site gluten detection because they offer a simple, on‐site format that requires no laboratory infrastructure. Results are typically available within 10–15 min, making them suitable for environmental monitoring, raw material screening, and production line verification. LFDs use antibodies such as R5, 2D4, or G12 and operate on the same antigen‐recognition principles as ELISA. Reported detection limits range from 5 to 10 mg/kg, although the food matrix can influence results. Simple matrices such as rinse water or swab extracts generally yield more reproducible signals than complex food matrices, although variability in swab extracts has also been documented depending on sampling technique and surface type (Amnuaycheewa et al. 2022; Scherf and Poms 2016). LFDs provide qualitative rather than quantitative results and are therefore unsuitable for regulatory compliance testing of final products. Some LFD formats provide qualitative or semiquantitative results through visual interpretation, while others use a dedicated reader to generate a numerical signal. Combining these validated qualitative LFDs for screening and reader‐based LFDs for higher‐resolution triage can support gluten management by flagging samples that require confirmatory laboratory testing near decision thresholds. They still serve as valuable tools in preventive gluten control programs. Guidance on the appropriate use of LFDs in gluten management programs is also being developed, including draft end‐user guidance from AOAC (AOAC Gluten and Food Allergens End‐User Guidance Working Group 2026). Many manufacturers integrate LFDs into prerequisite programs (PRPs) that underpin hazard analysis and critical control point (HACCP) systems, enabling early detection and corrective action before critical control points are reached. Validation procedures for LFDs are less harmonized than those for laboratory‐based methods—albeit recent guidance, including AOAC SG 2025.002, is beginning to address this gap—and comparative studies have shown variability between brands, particularly for thermally processed foods (C. Diaz‐Amigo and Popping 2012; Scherf and Poms 2016). For this reason, positive or borderline LFD results should be verified using validated quantitative methods such as ELISA or LC–MS/MS (Fernández‐Gil et al. 2021).

#### 3.1.2 Mass Spectrometry‐Based Methods

LC–MS/MS has become an important tool in gluten analysis, particularly for complex or processed foods where immunoassays may not provide reliable results. Unlike ELISA, LC–MS/MS identifies specific gluten‐derived peptides, enabling the direct detection of fragments that persist after processing and may no longer be recognized by antibodies. These peptide‐based measurements provide molecular confirmation of the cereal source and the presence of gluten in fermented or hydrolyzed products. Typical LC–MS/MS workflows involve protein extraction, enzymatic digestion (often with trypsin), and chromatographic separation, followed by multiple reaction monitoring (MRM). Selected peptides serve as markers for different gluten proteins from various gluten‐containing cereal sources. Marker peptides must be selected carefully, as small sequence modifications (e.g., single amino acid substitutions, deletions, insertions, posttranslational modifications, or food‐processing‐dependent alterations) can render the peptide undetectable (Alves et al. 2019). Method specificity depends on the choice of marker peptides (Lexhaller et al. 2019). LC–MS/MS is a relevant analytical approach for characterizing gluten proteins in candidate reference materials (Lexhaller et al. 2019).

Gluten quantitation by LC–MS/MS can be achieved using isotopically labeled peptides. For example, Xu et al. (2025) developed a protocol using four labeled peptides—two from α‐gliadin, one from γ‐gliadin, and one from a low‐molecular‐weight glutenin subunit—to quantify gluten in wheat‐based products. Their method could detect and quantify gluten below the regulatory 20 mg gluten/kg threshold and even detect gluten at approximately 10 mg gluten/kg, based on the best reporter maker. Stable isotope‐labeled internal peptides can also help correct for matrix effects.

Recent advances in the field, including work from the European ThRAll project, have strengthened the methodological foundation of LC–MS/MS in allergen detection and also contributed to the development of harmonized calibration protocols and performance benchmarks (Henrottin et al. 2023; Mills et al. 2023). Although not focused on gluten, its work on peptide selection and method standardization can also inform gluten‐specific assays. These efforts have demonstrated that LC–MS/MS can provide reliable detection of food allergens in processed matrices where ELISA performance declines. A key strength of LC–MS/MS is its ability to detect residual gluten peptides in samples exposed to heat, enzymatic hydrolysis, or fermentation. Studies have reported the presence of gluten peptides in enzyme‐treated beers that tested negative by ELISA (Tissen et al. 2025). In such cases, mass spectrometry offers a more detailed picture of hydrolyzed gluten products. Still, the immunotoxic potential of residual peptides and the interpretation of measured gluten peptides in these types of products remain challenging (Cubero‐Leon et al. 2024).

Despite these advantages, LC–MS/MS has limitations. The technique requires specialized instrumentation, trained personnel, and tight control of each analytical step from digestion and extraction to peptide quantification. The absence of a standardized panel of marker peptides and reference materials continues to restrict comparability across laboratories. Lexhaller et al. (2019) noted that this lack of harmonization parallels the calibration issues encountered in immunoassays. An additional limitation relates to quantification. LC–MS/MS measures selected marker peptides rather than total gluten protein. Conversion of peptide concentrations to regulatory gluten values expressed in milligrams per kilogram requires defined reference proteins, appropriate conversion factors, and assumptions regarding protein composition and digestion efficiency. These factors vary between wheat, rye, and barley and may change with processing, complicating direct comparison with immunoassay‐based results (Lexhaller et al. 2019; Tissen et al. 2026). Harmonization will also require uniform conversion factors for wheat, rye, and barley. Multi‐laboratory validation using agreed protocols and performance criteria will be required before LC–MS/MS can be adopted for routine regulatory testing. Current results are encouraging: state‐of‐the‐art methods achieve detection limits in the 5–10 mg gluten/kg range and quantify gluten reliably above the 20 mg gluten/kg threshold (Xu et al. 2025). LC–MS/MS, therefore, represents a strong candidate for confirmatory analysis in gluten compliance testing.

#### 3.1.3 Genomic and Emerging Detection Technologies

Molecular methods such as PCR and quantitative PCR (qPCR) detect DNA from gluten‐containing cereals, serving as proxies for gluten presence. As long as DNA remains intact, these techniques can confirm the presence of wheat, rye, or barley even when proteins have been denatured beyond recognition. PCR‐based methods cannot reliably quantify gluten as the relationship between DNA and gluten proteins is variable, and are therefore unsuitable for assessing compliance with gluten thresholds. In addition, processing and the characteristics of food matrices can affect DNA integrity; for example, acidic products degrade DNA, while gluten proteins and peptides may remain stable and detectable. Food processing can further affect PCR performance because DNA integrity and extraction efficiency depend on particle size distribution, matrix composition, and thermal treatment, which may bias quantitative PCR results in processed foods (Engel et al. 2006; Moreano et al. 2005). Several studies have compared ELISA and PCR methods in different samples, including raw materials and processed or incurred foods, which report comparable results or even better sensitivity (Mujico et al. 2011) and lower false positive rates (Henterich et al. 2003; Scharf et al. 2013) of PCR tests compared to their immunoassay counterparts.

Other detection technologies are at earlier stages of development and are not yet commercially available for routine use. Optical and electrochemical biosensors using immobilized antibodies or aptamers have achieved detection limits comparable to or lower than those of ELISA (Amaya‐González et al. 2014; Pinto et al. 2014; Tsai et al. 2024). Their potential for rapid, on‐site application makes them attractive for industrial quality control. Multiplex systems that integrate several allergen‐ and gluten‐specific antibodies on a single platform have been developed (Angelopoulou et al. 2018; Rallabhandi et al. 2020). This assay type has the potential to expand epitope coverage across cereal types. While promising, these approaches still require formal validation before they can be applied in regulatory contexts.

### 3.2 Extraction Procedures and Matrix Effects

Extraction is a critical step in gluten analysis, and it has been shown to be more important than gluten composition for ELISA detection of these cereal proteins in baked food products (Schirmer and Scherf 2023). The goal of the extraction process is to solubilize prolamins and glutelins. In antibody‐based methods, the procedure must avoid damaging the antigenic regions of the proteins required for antibody recognition.

The first gluten antibody‐based methods used gluten extraction with aqueous alcohol, usually 40%–70% ethanol, which was efficient only for extracting gluten proteins from minimally processed foods (C. Diaz‐Amigo and Popping 2012). However, this solution did not perform efficiently in processed foods. Reduced gluten solubility in processed samples requires effective sample extraction methods that often require harsher conditions. This situation led to the search for new extraction strategies better suited for treated food, such as baked products. A new generation of extraction solutions marked a turning point in gluten testing of processed samples. These solutions facilitate the solubilization of proteins (Garcia et al. 2005; Gessendorfer et al. 2010; Van Den Broeck et al. 2009) by incorporating reducing agents (e.g., 2‐mercaptoethanol or dithiothreitol) that break disulfide bonds and detergents or chaotropic agents (e.g., guanidine hydrochloride) that increase protein release (Güven and Azizoglu 2023). Examples include the cocktail solution (Doña et al. 2008), the universal prolamin and glutelin extractant solution (UPEX) (Mena et al. 2012), the universal gluten extraction solution (UGES) (Segura et al. 2021), and others (Gessendorfer et al. 2010). However, harsh sample extraction conditions can negatively affect antibody integrity and binding but typically do not adversely affect LC–MS/MS detection. In contrast, incomplete extraction leads to underestimation of gluten content. Balancing the use of harsh extraction conditions and antibody integrity is a key challenge in assay design, particularly when results are used to determine compliance with the 20 mg gluten/kg labeling threshold. Despite the progress, a recent study indicated that solutions commonly used in ELISA kits cannot completely extract gluten proteins from baked products (Schirmer and Scherf 2023).

Matrix composition is another factor influencing extraction efficiency and antibody binding. High‐fat or high‐sugar foods can limit solvent access to proteins, while fibrous or dense matrices may physically entrap gluten, making it less accessible to antibodies. Consequently, recovery rates vary widely between product types. Baked goods, hydrolyzed sauces, and extruded cereals often yield lower recoveries than flours or starches (DLA 2024; Food Standards Agency 2023; Rzychon et al. 2017). Robust validation must account for this variability by including a range of representative matrices (AOAC International 2025).

### 3.3 Reference Materials and Calibration

Gluten quantification relies on assay calibration against suitable reference materials. The PWG developed the PWG gliadin material (gliadin reference material), a wheat gliadin‐enriched fraction obtained from 28 European wheat cultivars (Van Eckert et al. 2006), although it is known to contain some glutelin contamination (Schalk et al. 2017). The PWG material is used to calibrate and standardize many commercial ELISA kits and has improved calibration comparability for wheat gliadin. However, it largely excludes glutelins (Schalk et al. 2017) and does not represent the full gluten protein profile of processed foods or nonwheat cereals. This difference can introduce bias, especially when testing products made from rye, barley, or mixed cereal sources (Lexhaller et al. 2016; Scherf and Poms 2016). There have been other initiatives and approaches to produce reference materials for gluten testing. These include mixtures of gluten protein fractions from wheat, barley, and rye (Schalk et al. 2017), a material consisting of a blend of flours from different wheat cultivars instead of purified gluten fractions (Hajas et al. 2018; Schall et al. 2020), or a material composed of hydrolyzed prolamins from wheat, rye, and barley for the quantification of partially hydrolyzed gluten (Gessendorfer et al. 2009). Nonwheat materials have also been proposed as secalins from seven rye cultivars (Xhaferaj et al. 2025) and a barley gluten material from eight different barley cultivars (Xhaferaj et al. 2025). Commercial wheat gliadins and gluten materials from different vendors have been evaluated for their potential use as reference materials (Xhaferaj and Scherf 2024). However, the limited representativeness of their protein composition and the batch‐to‐batch variability of these materials make them unsuitable candidates, highlighting the importance of properly characterized materials. A different approach, taking into consideration the food matrix, is the use of incurred materials containing gliadin as an alternative for the quantification of gluten in processed foods (Bugyi et al. 2012). Incurred materials better reflect the composition and structure of the food samples typically consumed, making them more suitable for method validation and proficiency testing (PT). One limitation of these materials is batch reproducibility, as it is challenging to produce materials with exactly the same attributes due to variations in gluten characteristics across wheat production seasons and locations. However, the use of blended flour helps reduce analytical error caused by genetic and harvest‐year variability (Schall et al. 2020). Until such materials become readily available, laboratories must rely on calibrants that represent only a subset of gluten proteins (e.g., gliadin‐based calibrants) or on matrix‐specific calibrants, which reduces comparability between analytical methods and testing environments.

### 3.4 Method Validation and Reproducibility

Validated analytical methods are essential for producing reliable and reproducible gluten measurements. Validation assesses key performance characteristics, including sensitivity, quantification limits, linearity, recovery, precision, and robustness across different food matrices. In gluten testing, these studies should use incurred samples rather than spiked controls to reflect the matrix effects introduced by processing and formulation. The AOAC Standard Guidance SG 2025.002 on Validation of Quantitative Gluten Methods (AOAC International 2025) emphasize the importance of inter‐matrix validation and the statistical evaluation of repeatability and reproducibility. Most published validation studies are conducted within a single laboratory and cover only a limited range of food categories, describing internal precision rather than the measurement uncertainty that interlaboratory PT can reveal.

Participation in PT provides external confirmation of laboratory competence. Providers such as FAPAS and DLA distribute incurred samples, allowing comparison between laboratories and, with sufficient participation, between analytical methods. Results repeatedly show measurement variability near the 20 mg gluten/kg threshold, particularly in processed foods. These findings underline the need for harmonized performance criteria to guide method selection, validation, and data interpretation.

### 3.5 PT and Interlaboratory Comparisons

PT is a key element of external quality assurance for laboratories performing gluten and other allergen analyses. It allows comparison of analytical results across laboratories analyzing identical samples under standardized conditions. PT serves two main functions: verifying the technical competence of participating laboratories and identifying methodological or matrix‐specific factors that influence gluten quantification across different testing environments. PT is particularly important in allergen detection, where results form the basis for regulatory decisions linked to strict gluten thresholds such as the 20 mg gluten/kg limit. Even validated ELISA kits can yield different results because of variations in antibody specificity, extraction efficiency, or matrix interactions. Participation in PT rounds provides an external benchmark for laboratories to evaluate the reliability of their methods, their competence in performing the method, and the presence of bias, variable recovery, or matrix effects that may not be visible in routine internal quality controls.

Dedicated gluten PT schemes are operated by organizations such as the German DLA – Proficiency Tests GmbH and the UK‐based FAPAS. Both are accredited under ISO 17043 and use incurred test materials that reflect realistic analytical challenges. Their cumulative datasets document long‐term trends in interlaboratory reproducibility and provide evidence of matrix‐dependent variability (Grundy et al. 2023; Owen and Gilbert 2009; Senyuva et al. 2019; Sykes et al. 2012).

#### 3.5.1 DLA PT Scheme for Gluten Analysis

Since 2006, DLA has included gluten detection as a key component of its broader allergen PT programs, progressively refining its design and analytical scope. In the original format, each PT round included two samples: a naturally contaminated positive sample and a matched negative matrix of the same composition. The positive samples were commercial food products known or suspected to contain gluten, while the negative samples were selected for similar composition and confirmed to have gluten levels below the ELISA's limit of detection (LOD). Because spiked samples were used only in a few cases, gluten concentrations in the positive samples were either unknown or estimated from supplier data. In 2013, DLA extensively reviewed PT samples to which a known amount of gluten was added and identified challenges with recovery rates and accurate gluten quantification (Scharf et al. 2013). Some test kits, despite fair recovery rates, received poor *z*‐scores (Andrade 2021; Kraeuter 2023; Moeller 2025), whereas other methods—some of which dominated the PT consensus—showed comparatively low recoveries but still produced favorable *z*‐scores due to their alignment with the consensus mean. PT performance is commonly expressed as a *z*‐score, which reflects deviation from the assigned value relative to the scheme's target standard deviation (ISO 2022). Because the assigned value is often derived from participants’ consensus, acceptable *z*‐scores indicate agreement within the PT scheme but do not necessarily demonstrate analytical trueness. This discrepancy highlighted that *z*‐scores alone could be misleading indicators of method performance, particularly when consensus‐based statistics disproportionately reflected the dominant methods rather than true recovery accuracy. However, the influence of matrix effects and processing could not be determined, as the unprocessed starting material was unavailable in these PTs.

These findings indicated that, although the early PT concept was practical and still widely used, an approach was required that assessed method performance against assigned values derived from defined spiking levels, enabling a more objective evaluation across different matrices and processing conditions (Scharf et al. 2013).

In 2014, DLA therefore introduced a third sample type, the spiking‐level sample, an unprocessed low‐interference reference matrix spiked with the same gluten material as the positive matrix sample, transforming the scheme from the typical purely comparative format into one with the potential of recovery rate estimation.

In this configuration, the positive‐matrix sample (e.g., cookies, chocolate, ice cream, rice cracker, infant formula, soy milk, or sausage) was spiked in‐house and, in some cases, further processed (baked, heated, cooked, or frozen). The same spiking material was also incorporated into a low‐interference, unprocessed matrix, typically potato powder, initially at higher concentrations and, since 2017, at an almost identical nominal gluten concentration. This design enabled participating laboratories and scheme organizers to estimate recovery and evaluate the combined influence of matrix effects and processing. The spiking‐level sample thus served as a reference under near‐ideal recovery conditions, because it used an unprocessed low‐interference matrix with limited co‐extractives that can reduce protein solubility or interfere with antibody binding, providing a benchmark against which performance in more complex or thermally processed samples could be assessed. This approach aligns with the principles outlined in the AOAC Guidelines for Validation of Quantitative Gluten Methods (AOAC International 2025), which emphasize the necessity of inter‐matrix validation.

This further standardized format enabled direct sample‐to‐sample comparison within and between laboratories. Since 2018, this three‐sample format—comprising a negative matrix, a positive matrix, and a spiking‐level sample—has been accredited under ISO 17043. Method‐dependent variation remains a key analytical challenge, particularly with ELISA‐based methods. Although ELISA remains the dominant tool in routine testing, its response varies with matrix composition and processing intensity. Additional variability arises from differences between commercial ELISA kits, including antibody specificity and extraction buffer formulation. To manage this, DLA evaluates results from each PT round both collectively and by analytical method. The collective assigned value (XALL) is calculated from all participant results regardless of the analytical kit used. In addition, method‐specific assigned values (Xmethod) are calculated for individual ELISA kits when at least five laboratories report results using the same kit. Because the RIDASCREEN Gliadin ELISA R7001 is typically the most widely used method in the scheme, its method‐specific assigned value (XRS) can serve as a reference. For any ELISA kit with at least five participating laboratories, a separate assigned value is calculated (consensus X method). The RIDASCREEN Gliadin ELISA R7001 is typically the most widely represented, with 47%–94% of participants per round, providing a robust dataset for assessing method‐specific trends.

Between 2017 and 2025, 17 PT rounds were conducted using this three‐sample design. All positive samples were spiked with a composite premix of 21 wheat flours sourced from different regions, yielding gluten concentrations ranging from 14 to 56 mg gluten/kg. The average gluten content for the matrix and spiking‐level samples was 28 and 27 mg gluten/kg, respectively, ensuring consistency for recovery evaluation. Recovery in unprocessed spiking‐level samples consistently exceeded 100%, with mean values of 142% for XALL and 134% for XRS. These elevated values likely reflect the favorable characteristics of the test matrix—low in co‐extractives, inhibitors, and processing‐related interferences—though the influence of matrix‐specific factors such as protein–matrix interactions and extraction–solvent compatibility cannot be entirely excluded. Similar overrecovery occurred in unprocessed infant food powders, where average recoveries reached 150% (XALL) and 147% (XRS). Matrix composition can increase or decrease apparent recovery, depending on its effects on protein extractability and assay response. In these low‐interference powders, high extractability and strong assay response may contribute to elevated values. In contrast, in other matrices, the same factors can contribute to underrecovery through binding, masking, or reduced solubility. Even relatively minor matrix modifications affected recovery. In one round, adding 15% strawberry powder reduced the gluten signal by 40%, possibly through signal suppression or impaired extractability. Likewise, preparing infant porridge with 50°C water resulted in a 14% decrease in signal, suggesting that even mild thermal treatment influences recovery. Heat exposure had a more pronounced impact on baked products. Lightly baked matrices, such as crispbread and rice crackers (190°C for 30 min), showed 10% lower recovery than the corresponding spiking‐level sample when normalized to each sample's nominal spiking level. In contrast, biscuits baked at 150°C for 40 min showed moderate reductions—12% (XALL) and 15% (XRS). More heavily baked matrices such as bread and cake (150°C–190°C for 60–90 min) exhibited reductions of 35%–38%, consistent with the hypothesis that prolonged heating denatures gluten or decreases its solubility, thereby lowering antibody recognition. In high‐fat or thermally intense matrices, similar losses were observed. Sausages processed at 100°C for 60 min showed recoveries of 31% (XALL) and 23% (XRS), both lower than their spiking‐level counterparts. Dark chocolate processed at 40°C–50°C for more than an hour yielded reductions of 35% (XALL) and 25% (XRS). Although absolute values differed, the trend was consistent: increasing thermal load, fat content, and overall matrix complexity reduced protein extraction and ELISA signal strength even when the same gluten source was used (Table 1).

**TABLE 1:** DLA gluten proficiency test (PT) samples used in the manuscript, showing the test matrix, processing conditions (where applicable), and gluten mass fraction (mg/kg). “Spiked or incurred gluten” refers to the assigned value for the PT item (provider characterization). “Spiking‐level sample gluten” refers to the nominal target level used when preparing the material, where reported. Differences between these columns can occur because the assigned value is based on the characterization of the final PT item and may differ from the nominal preparation level. A dash indicates not reported.

PT no.	Food matrix samples	Processing	Spiked or incurred gluten (mg/kg)	Spiking‐level sample gluten (mg/kg)
DLA 03/2018	Infant food, cereal pap powder, gluten‐free	—	37.7	36.1
ptAL03 (2023)	Infant food, soy formula powder, gluten‐free	—	17.4	17.5
ptAL03 (2021)	Infant food, soy formula powder with hydrolyzed milk protein, gluten‐free	—	15.1	14.7
DLA 03/2017	Infant food, cereal pap powder, gluten‐free	—	56.4	47.8
ptAL03 (2022)	Infant food, cereal pap powder, gluten‐free	—	22.5	23.8
ptAL03 (2024)	Infant food, hydrolyzed milk protein formula, powder, gluten‐free	—	21.0	21.6
DLA 03/2019	Infant food, cereal pap powder, gluten‐free	—	31.9	36.2
ptAL02 (2021)	Crisp bread, baked	195° / 30 min	41.4	20.7
ptAL02 (2023)	Rice cracker, baked	190° / 30 min	13.7	17.8
ptAL02 (2024)	Bread baking mix	—	22.6	22.0
ptAL03 (2020)	Infant food, cereal pap prepared with water, gluten‐free	50°C	22.1	19.9
ptAL02 (2020)	Cookies, baked	150° / 40 min	18.1	35.6
ptAL02 (2025)	Sausage, boiled	100°C / 60 min	31.7	37.9
ptAL06 (2022)	Dark chocolate, warmed	40‐50°C / >60 min	22.3	22.0
ptAL02 (2022)	Marble cake, baked	150° / 60 min	17.7	16.9
DLA 02/2019	Bread, baked, gluten‐free	190° / 90 min	50.6	30.4
ptAL03 (2025)	Infant food, cereal pap with fruit, powder gluten‐free (15% strawberry powder)	—	31.1	30.6
ptAL11 (2023) B	Oat flour	—	17.4	—
ptAL11 (2023) D	Rice flour	—	17.5	—
ptAL10 (2023) B	Oat drink, heated	70°C / 10 min	18.6	—
ptAL10 (2023) D	Rice drink, heated	70°C / 10 min	20.5	—

**FIGURE 1:**
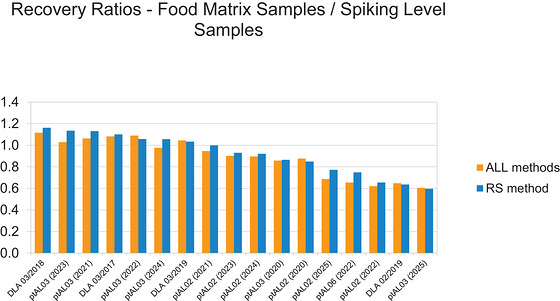
Recovery ratios in DLA gluten proficiency testing across different food matrices. Ratios represent the measured gluten concentration in the matrix sample relative to the nominal gluten level in the corresponding spiking‐level sample. ALL methods (XALL) show the consensus value calculated from all reported analytical methods, while the RS method (XRS) shows the consensus value calculated from results obtained with the RIDASCREEN Gliadin ELISA R7001.

Additional PT rounds in 2023 examined plant‐based matrices. Gluten‐free oat and rice flours, and their corresponding beverages processed at 70°C for 10 min, were assessed. In oat‐based samples, beverage formats gave signals 18% (XALL) and 13% (XRS) lower than the flour; in rice‐based products, the reductions were 32% (XALL) and 23% (XRS). These differences suggest that protein dispersion and moderate heating reduce extractable gluten in liquid matrices. Fermented matrices, particularly beer, posed a separate analytical challenge. Although PT rounds for beer were excluded from quantitative recovery analysis due to the unknown initial gluten content, they still provided useful insights. In all beer‐focused rounds, the RIDASCREEN Gliadin Competitive ELISA R7021 was the most frequently used method and consistently reported the highest gluten values, especially for barley malt samples. Because these products lack a defined gluten baseline, quantitative interpretation is limited, but the data confirm the difficulty of detecting hydrolyzed gluten with sandwich‐type ELISAs (Figure 1).

In hindsight, inclusion of a spiking‐level sample has been crucial in benchmarking recovery. It enables laboratories to distinguish between limitations inherent to the analytical method and those arising from matrix composition or processing. Significant deviations from expected recovery—whether excessive or deficient—indicate a need to reassess extraction conditions or to apply complementary detection methods tailored to the specific matrix.

#### 3.5.2 FAPAS PT Scheme—Data Evaluations and Observations

FAPAS, a UK‐based provider accredited under ISO 17043, operates allergen PT schemes that separate results by ELISA kit. This acknowledges the known variability between commercial assays, where differences in antibody specificity, extraction chemistry, and calibration procedures can influence reported gluten concentrations. Unlike most food PT programs that assign target values from a collective mean, FAPAS considers kit‐specific performance when calculating assigned values. Both DLA and FAPAS evaluate results by analytical method in addition to the collective assigned value, acknowledging that kit‐specific factors—including antibody specificity, extraction chemistry, and calibration materials, particularly when calibrants are not matrix‐matched—can introduce systematic bias and substantially influence reported gluten concentrations and recovery estimates. This distinction is particularly relevant in gluten testing, where even minor procedural differences can produce substantial variation in measured levels.

Between 2022 and 2025, FAPAS evaluated seven gluten PT rounds across 11 datasets, including common food matrices such as flours, baked goods, and high‐fat products (Table 2).

**TABLE 2:** Summary data of FAPAS gluten PTs showing assigned values (AV, mg gluten/kg) reported separately by ELISA kit and as “All data combined” for each PT item. *n* is the number of results included in the calculation. “#” indicates an assigned value reported for information only. All data combined AV provides a within‐PT summary across all reported methods for that PT item and supports comparisons between kits within the same PT round. Comparisons of AVs between different PT references or years should be interpreted in light of differences in matrix and gluten level between PT items.

PT reference	Year	Matrix	Analyte (kit or data type)	*n*	AV (mg gluten/kg)
27331A	2022	Cake mix	Romer Labs AgraQuant ELISA Gluten G12 (COKAL0200)	8	27.2
27331A	2022	Cake mix	SENSISpec Ingezim Gluten R5 (30.GLU.K.2)	7	13.6
27331A	2022	Cake mix	Neogen Veratox for Gliadin R5 (8510)	19	15.0
27331A	2022	Cake mix	R‐Biopharm RIDASCREEN Gliadin (R7001)	88	13.9
27331A	2022	Cake mix	R‐Biopharm RIDASCREEN Fast Gliadin (R7002)	7	13.8
27331A	2022	Cake mix	All data combined	142	13.9
27331B	2022	Cake mix	Romer Labs AgraQuant ELISA Gluten G12 (COKAL0200)	8	17.5
27331B	2022	Cake mix	SENSISpec Ingezim Gluten R5 (30.GLU.K.2)	7	5.03
27331B	2022	Cake mix	Neogen Veratox for Gliadin R5 (8510)	13	6.00
27331B	2022	Cake mix	R‐Biopharm RIDASCREEN Gliadin (R7001)	48	5.53
27331B	2022	Cake mix	All data combined	90	5.41
27346	2023	Cooked biscuit	SENSISpec Ingezim Gluten R5 (30.GLU.K.2)	3	12.2
27346	2023	Cooked biscuit	Neogen Veratox for Gliadin R5 (8510)	12	24.9
27346	2023	Cooked biscuit	R‐Biopharm RIDASCREEN Gliadin (R7001)	58	21.7
27346	2023	Cooked biscuit	R‐Biopharm RIDASCREEN Fast Gliadin (R7002)	5	19.0
27346	2023	Cooked biscuit	All data combined	85	20.2
27350A	2023	Infant formula	SENSISpec Ingezim Gluten R5 (30.GLU.K.2)	6	17.3
27350A	2023	Infant formula	Neogen Veratox for Gliadin R5 (8510)	5	20.0
27350A	2023	Infant formula	R‐Biopharm RIDASCREEN Gliadin (R7001)	21	21.0
27350A	2023	Infant formula	All data combined	38	19.8
27377A	2024	Cake mix	SENSISpec Ingezim Gluten R5 (30.GLU.K.2)	10	8.98
27377A	2024	Cake mix	Neogen Veratox for Gliadin R5 (8510)	4	9.63
27377A	2024	Cake mix	R‐Biopharm RIDASCREEN Gliadin (R7001)	35	10.5
27377A	2024	Cake mix	All data combined	62	9.93
27377B	2024	Cake mix	SENSISpec Ingezim Gluten R5 (30.GLU.K.2)	10	38.6
27377B	2024	Cake mix	R‐Biopharm RIDASCREEN Gliadin (R7001)	35	39.3
27377B	2024	Cake mix	All data combined	63	40.1
27386B	2024	Infant soya formula	Neogen Veratox for Gliadin R5 (8510)	4	20.1
27386B	2024	Infant soya formula	R‐Biopharm RIDASCREEN Gliadin (R7001)	19	23.1
27386B	2024	Infant soya formula	SENSISpec Ingezim Gluten R5 (30.GLU.K.2)	4	15.3 #
27386B	2024	Infant soya formula	All data combined	38	20.8
27395A	2024	Cake mix	Neogen Veratox for Gliadin R5 (8510)	7	18.0
27395A	2024	Cake mix	R‐Biopharm RIDASCREEN Gliadin (R7001)	71	19.4
27395A	2024	Cake mix	SENSISpec Ingezim Gluten Quick (30.GL2.K.2)	4	17.6 #
27395A	2024	Cake mix	SENSISpec Ingezim Gluten R5 (30.GLU.K.2)	5	18.6
27395A	2024	Cake mix	All data combined	92	18.5
27418 allergenic protein	2025	Cooked biscuit	allergenic protein	19	22.2
27418 ELISA	2025	Cooked biscuit	R‐Biopharm RIDASCREEN Gliadin (R7001)	53	25.2
27418 ELISA	2025	Cooked biscuit	Romer Labs AgraQuant ELISA Gluten G12 (COKAL0200)	3	30.5
27418 ELISA	2025	Cooked biscuit	SENSISpec Ingezim Gluten R5 (30.GLU.K.2)	6	17.3 #
27418 all	2025	Cooked biscuit	All data combined	98	23.9

**FIGURE 2:**
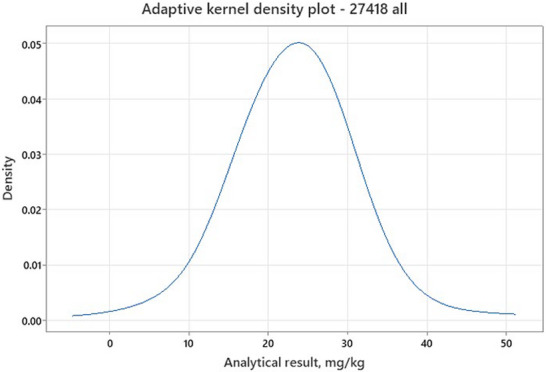
Kernel density plot of all gluten results in PT 27418, including allergenic protein fraction and ELISA results. Kernel density plot of reported results for FAPAS PT 27418 (cooked biscuit), combining ELISA results and results reported under the PT category “allergenic protein fraction” (non‐ELISA). All values are expressed as milligrams gluten per kilogram gluten, as reported by the PT provider, and are plotted together to show the overall distribution of participant results.

**FIGURE 3:**
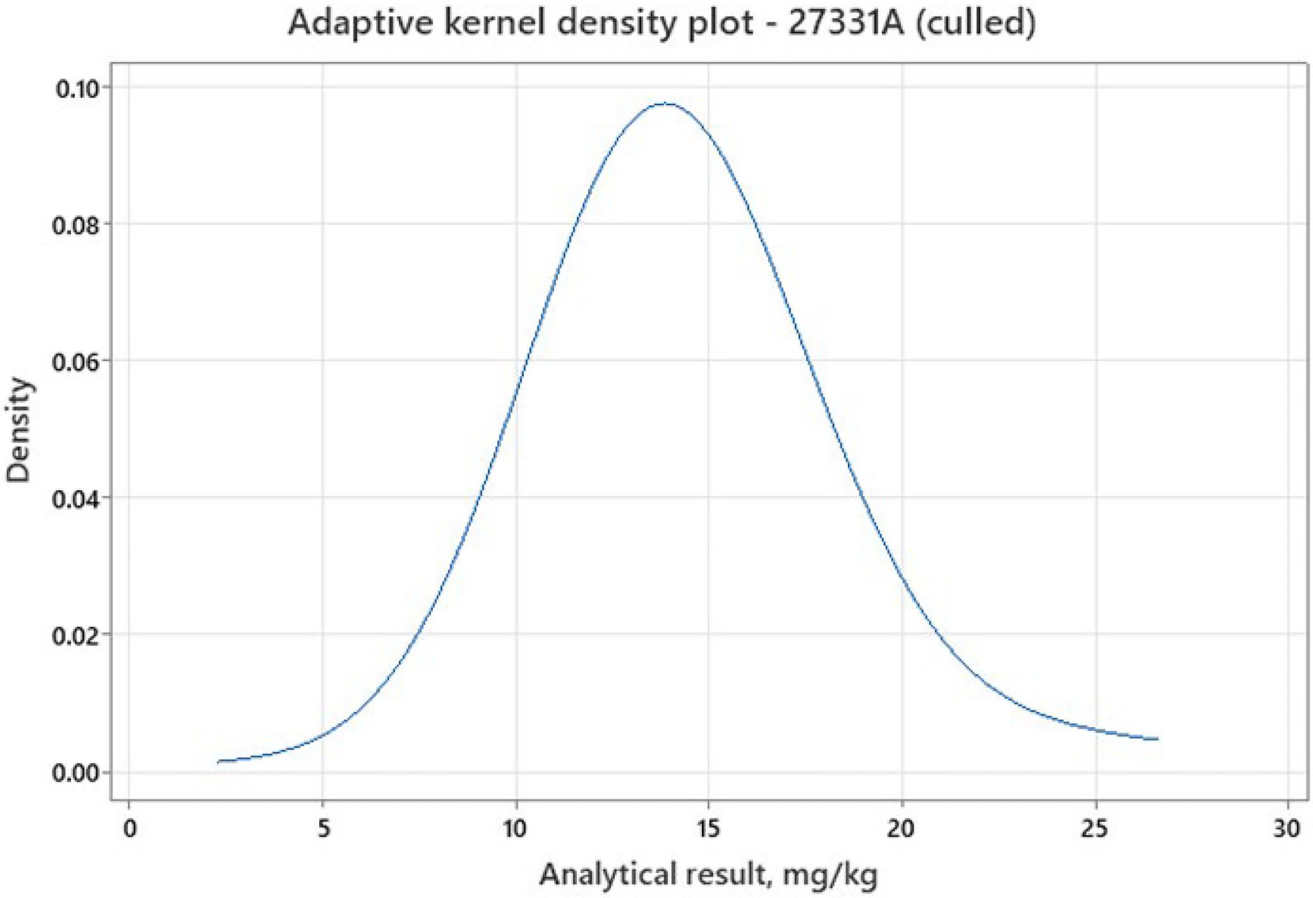
Kernel density plot of all gluten results in PT 27331 sample A, excluding one extreme outlier result.

Statistical analyses applied kernel density estimation (KDE) to detect potential clustering of results by analytical method. The combined datasets showed unimodal, symmetrical distributions across rounds, indicating broad convergence in performance between ELISA kits. In most cases, assigned values for different kits were closely aligned. The uncertainty‐to‐proficiency ratio (*u*/*σ*pt) ranged from 0.07 to 0.20—well below the acceptance limit of 0.3—confirming that *z*‐scores could be confidently assigned to all participants (Table 3; Figures 2 and 3).

**TABLE 3:** Assigned values (AV) calculated as the mode by kernel density estimation for all results within a PT, with standard error of the mode (sem) calculated by the bootstrap. *u* is the observed uncertainty (equivalent to sem), and *σ*pt is the standard deviation for proficiency assessment.

PT reference	Year	Matrix	AV (mg gluten/kg)	sem	*u*/*σ*pt
27331A	2022	Cake mix	13.9	0.244	0.0702
27331B	2022	Cake mix	5.41	0.118	0.0872
27346	2023	Cooked biscuit	20.2	0.810	0.160
27350A	2023	Infant formula	19.8	0.862	0.174
27377A	2024	Cake mix	9.93	0.489	0.197
27377B	2024	Cake mix	40.1	0.870	0.0868
27386B	2024	Infant soya formula	20.8	0.968	0.186
27395A	2024	Cake mix	18.5	0.827	0.179
27418 all	2025	Cooked biscuit	23.9	0.932	0.156

A notable exception occurred in two PT rounds, in which laboratories using the Romer Labs AgraQuant Gluten G12 kit reported concentrations roughly twice those of other participants. These results represented less than 10% of total submissions and therefore did not compromise the statistical consensus. They illustrate how calibration or antibody differences can still affect absolute quantification, even under harmonized PT conditions.

Overall, the evaluation points to an emerging alignment in gluten ELISA performance across commercial kits. This trend appears more pronounced in gluten ELISA kits than in those for allergens or intolerance markers, likely reflecting the strong regulatory and clinical focus on gluten testing. The need to comply with legally defined thresholds has driven more rigorous calibration, validation, and cross‐kit comparison efforts. Continued monitoring across additional food matrices and analytical conditions will be necessary to confirm whether this convergence persists as new methods enter the market.

#### 3.5.3 Summary of Key Insights From PT

PT data from DLA and FAPAS confirm that gluten analysis remains strongly influenced by matrix composition, even under controlled conditions. Interlaboratory agreement is generally high for unprocessed or starch‐rich samples, but rounds involving processed or thermally treated foods continue to show reduced recovery. Published provider reports (DLA 2025; FERA 2025) and comparative studies (Grundy et al. 2023; Owen and Gilbert 2009; Rzychon et al. 2017; Senyuva et al. 2019; Sykes et al. 2012) consistently demonstrate that ELISA‐based methods tend to underestimate gluten in fat‐rich, baked, or fermented products. These deviations arise mainly from incomplete extraction or epitope modification rather than procedural error. Importantly, acceptable *z*‐scores do not always indicate analytical accuracy, as many laboratories report concentrations well below the expected spiking level. FAPAS datasets show median recoveries 20%–40% below assigned values in high‐fat and baked matrices, while DLA reports signal losses of up to 35% in thermally processed foods. Such findings confirm that reliable gluten quantification across diverse food categories remains technically challenging despite improved validation protocols and greater availability of incurred reference materials.

Continued participation in accredited PT schemes remains key to ensuring analytical consistency. Testing under real‐world conditions helps laboratories identify systematic weaknesses related to antibody performance, extraction efficiency, or matrix composition. Over time, PT outcomes have contributed to refinements in validation design and the development of improved reference materials (AOAC International 2025; Holzhauser et al. 2020; Osorio et al. 2019). Comparative PT data complement in‐house controls by providing independent interlaboratory evidence of performance across matrices and processing conditions and remain central to maintaining confidence in gluten‐free labeling practices.

### 3.6 Analytical Reliability and Economic Implications

Variability in gluten test results has significant economic and regulatory implications for the gluten‐free sector. Analytical errors near the 20 mg gluten/kg threshold can lead to consumer exposure, product withdrawals, and liability claims, with consequences that extend beyond individual batches (Guennouni et al. 2022; Scherf and Poms 2016). Recent data illustrate these issues are widespread: approximately 4%–5% of meals served as gluten‐free in food service settings have been found to exceed the 20 mg gluten/kg threshold. Other surveys found even higher contamination rates in nonindustrial environments, ranging from 13% to 40%, depending on sampling strategy and product type (Silvester et al. 2016; Thompson 2020). These findings reflect a combination of inadequate process controls, environmental cross‐contact, and inconsistent analytical methods. In commercial production, variability in sampling and subsampling, extraction, and method performance can lead to costly product withdrawals, even when different laboratories report divergent results for the same production batch.

The financial impact of gluten‐related recalls is considerable. In the United States, the average direct cost per recall incident exceeds USD 10 million, excluding reputational losses or indirect costs such as lost contracts and market delays (GMA 2011). Although newer comprehensive cost estimates are limited, this figure illustrates the potential order of magnitude of recall‐related losses. Within the European Union, test inconsistencies have caused trade disputes (Amnuaycheewa et al. 2022) and created uncertainty for manufacturers seeking mutual recognition of gluten‐free claims. Manufacturers depend on reliable analytical data to demonstrate compliance to regulators, retailers, and certification bodies. Yet method‐dependent variation caused by differences in assay design (e.g., antibodies, extraction solvents, and calibration strategies) continues to yield divergent values near the 20 mg gluten/kg threshold (Lexhaller et al. 2016; Schopf and Scherf 2018). These discrepancies are most critical when decisions rely on marginal numerical differences. Without harmonized validation criteria and defined statistical limits for uncertainty and reproducibility, interpretation of results remains inconsistent across jurisdictions.

Some private certification schemes, such as the BRCGS Global Standard Gluten‐Free (BRCGS 2024), aim to mitigate this uncertainty by requiring accredited methods, verification procedures, and risk‐based sampling. In practice, many accredited gluten methods are based on the R5 Méndez ELISA or closely related assay formats, which provide a common analytical basis but can also introduce method‐dependent bias. These systems depend on the foundation of analytical comparability. Establishing shared validation criteria is essential to achieve regulatory consistency and maintain market confidence. Reproducible, matrix‐tested methods and agreed benchmarks for sensitivity, accuracy, and precision are key to ensuring consistent enforcement, credible certification, and effective consumer protection.

## 4 Regulatory and Standardization Context

The governance of gluten‐free labeling combines public regulation, international standardization, and private assurance frameworks. Together, these mechanisms define how products are manufactured, verified, and placed on the market with a gluten‐free claim. Legally binding regulations establish thresholds and labeling requirements, while private standards provide audit‐based systems that integrate analytical verification into routine compliance management.

### 4.1 International Regulatory Frameworks and the Role of CODEX

The main international reference for gluten‐free labeling is the Codex Alimentarius Standard CXS 118–1979 (FAO and WHO, n.d.), first issued in 1979 and last revised in 2015. The standard defines gluten‐free foods as those containing less than 20 mg gluten/kg in the final product. This definition applies both to foods made from inherently gluten‐free raw materials and to those derived from gluten‐containing cereals that have undergone a removal process. A second category covers foods specially processed to reduce gluten content, allowing up to 100 mg gluten/kg. This category is rarely applied in practice, as most jurisdictions align with the 20 mg gluten/kg threshold for gluten‐free labeling. The standard requires that analytical methods used for verification be validated and fit for their intended purpose. Codex gluten quantification is historically linked to the R5 Méndez ELISA as the Type I defining method for gluten‐free provision. This provides a common analytical reference point for enforcement. Still, comparability is, in practice, influenced by the behavior of that defining method, particularly when laboratories use assays based on different antibodies, extraction procedures, or calibration systems. For this reason, method performance criteria (MPCs) should support fitness‐for‐purpose across matrices while recognizing method dependency introduced by the defining method concept. Under the Codex framework, regulatory enforcement lies with national competent authorities, while food business operators are responsible for managing gluten risk throughout production and supply chains.

### 4.2 Regulatory Frameworks in Europe and Selected Countries

Building on Codex CXS 118–1979, a range of national and regional regulations have incorporated its thresholds into legally enforceable rules (Table 4).

**TABLE 4:** Regulatory gluten thresholds and labeling criteria in selected jurisdictions.

Authority/framework	Legal basis	Threshold (mg gluten/kg)	Model type	Notes
Codex	STAN 118–1979	≤20	Quantitative	Also “very low gluten” ≤100
EU	Reg. 828/2014	≤20	Quantitative	Optional ≤100 category
USA	21 CFR §101.91	<20	Quantitative	Includes fermented foods
Canada	FDR B.24.018	<20 (operational)	Quantitative	No explicit numeric in text
Australia/NZ	FSANZ Code	No detectable	Qualitative	Stricter than Codex
Argentina	CAA	≤10	Quantitative	Official symbol required
Chile	RSA	≤3	Quantitative	Ultra‐low threshold
Brazil	Law 10.674	No threshold	Presence/absence	Binary declaration

#### 4.2.1 European Union and National Regulations

Within the European Union, allergen ingredient labeling is governed by Regulation (EU) No 1169/2011 on the provision of food information to consumers. The specific rules for voluntary gluten‐free and very low gluten claims are defined in Commission Implementing Regulation (EU) No 828/2014, which incorporates the Codex threshold of 20 mg gluten/kg for foods labeled “gluten‐free” and 100 mg gluten/kg for foods labeled “very low gluten.” Official enforcement of these requirements is carried out under Regulation (EU) 2017/625 on official controls, which replaced Regulation (EC) No 882/2004 and establishes the framework for sampling, laboratory testing, and verification by competent authorities. Regulation 1169/2011 governs consumer food information and requires the mandatory declaration of gluten‐containing cereals—namely wheat, rye, barley, and oats—in both prepacked and non‐prepacked products. Oats, although tolerated by many coeliac patients, are classified as gluten‐containing unless produced under strictly controlled conditions that guarantee purity and gluten levels below 20 mg gluten/kg. Regulatory treatment of oats differs internationally. In some jurisdictions, including Australia and New Zealand, oats are considered part of the gluten‐containing cereal group for labeling purposes, and foods containing oats cannot be marketed as gluten‐free. In the European Union, Regulation (EU) No 1169/2011 also includes provisions for how allergen information is communicated in non‐prepacked foods. Article 44 allows Member States to define how allergen declarations are communicated in non‐prepacked contexts such as food service or catering.

Implementing Regulation 828/2014, adopted under Article 36(3)(d) of Regulation 1169/2011, sets the conditions for the voluntary use of the terms “gluten‐free” and “very low gluten.” These claims may be used on prepacked or non‐prepacked foods if the gluten level remains below 20 or 100 mg gluten/kg, respectively. The regulation distinguishes between inherently gluten‐free foods, such as rice or maize, and products that have undergone processing to reduce gluten, ensuring that labeling does not mislead consumers. In addition to labeling rules, European food law also requires preventive allergen management during food production. Regulation (EC) No 852/2004 on the hygiene of foodstuffs, Annex II, Chapter IX, point 9, requires food business operators to implement procedures that prevent cross‐contamination of foods with allergens during production, processing, and handling. These hygiene provisions support the reliability of voluntary gluten‐free claims by requiring manufacturers to control sources of unintended gluten contamination throughout the production chain.

Enforcement procedures are governed by Regulation (EU) 2017/625 on official controls (European Union 2017), which delegates authority for sampling and analysis to designated national agencies. Laboratories performing official gluten testing must be accredited to ISO/IEC 17025 (ISO 2021) and use validated methods with quantification limits appropriate for assessing compliance with the 20 mg gluten/kg threshold. In practice, guidance from SNE and AOAC recommends a limit of quantification (LOQ) of around 10 mg gluten/kg to ensure sufficient analytical sensitivity. Measurement uncertainty must also be considered when interpreting results close to the regulatory threshold.

The practical implications of enforcement extend beyond legal compliance to economic and reputational risks. Products found to exceed the 20 mg gluten/kg limit while bearing a gluten‐free label may be subject to mandatory recall, causing brand damage and legal exposure, especially where consumer safety is affected. False‐positive findings have their own costs: when analytical overestimation leads to the withdrawal of compliant products, manufacturers incur unnecessary recall costs, distribution delays, and loss of customer confidence. These risks are particularly significant for producers operating under third‐party certification schemes or exporting to markets with stringent gluten‐free requirements.

Food safety authorities across Europe have documented recalls of foods labeled gluten‐free due to gluten content exceeding regulatory thresholds. While published data rarely identify the root cause, analytical variability and matrix extraction challenges are recognized in the literature as potential contributing factors. The absence of harmonized interpretation criteria and defined tolerance ranges complicates enforcement when results fall near the threshold. The finding that approximately 4%–5% of meals advertised as gluten‐free in food service settings exceeded 20 mg gluten/kg is a telling indicator of how gaps in analytical verification translate directly into erosion of consumer confidence and regulatory trust. In contrast, FDA surveillance sampling of foods labeled gluten‐free in 2015–2016 reported a product‐based compliance rate above 99.5% with the 20 mg gluten/kg requirement, with noncompliance limited to a small number of samples from a single product source (FDA 2016).

Germany provides a detailed example of coordinated enforcement. The Federal Office for Consumer Protection and Food Safety (BVL) (BVL, n.d.c) oversees gluten control alongside two expert bodies: the ALS (Arbeitskreis Lebensmittelchemischer Sachverständiger) (BVL, n.d.a) and the ALTS (Arbeitskreis Lebensmittelhygiene und Lebensmittel tierischer Herkunft) (BVL, n.d.b). These committees issue binding technical opinions to harmonize enforcement across federal states. One such document, Joint Opinion 2019/58, describes how to interpret gluten findings in products labeled gluten‐free (“113. ALS‐Sitzung” 2019). It advises that a confirmed laboratory result above 20 mg gluten/kg may be considered potentially harmful under Article 14(2)(a) of Regulation (EC) No 178/2002 if the resulting daily intake exceeds about 50 mg of gluten. Although not legally binding, this guidance is universally applied in Germany. This guidance uses an exposure‐based interpretation by linking confirmed analytical results to an estimated daily gluten intake. Because approaches of this type differ between countries and are not harmonized, they can reduce comparability between national enforcement practices and published analytical datasets.

A similar approach exists in Austria, where the “Not Safe” working group, led by the competent authority, has developed assessment criteria for interpreting gluten analyses. According to Appendix 6 of the guideline “Foods labeled gluten‐free or very low gluten content—Not Safe,” products declared as gluten‐free with a gluten content above 20 mg gluten/kg are regarded as misleading. The same applies to foods labeled as having very low gluten content if they exceed 100 mg gluten/kg. These thresholds apply only to foods marketed with gluten‐free or very low gluten claims. Products that intentionally contain gluten‐containing cereals and declare them as ingredients may exceed these levels; however, foods containing more than 20 mg gluten/kg cannot be labeled gluten‐free regardless of whether the gluten originates from ingredients or from unintended cross‐contact. In both cases, exposure to more than 50 mg of gluten per day is assessed as “unsafe—harmful to health,” reflecting the same risk basis applied in Germany.

Before enforcement actions, such as product withdrawal or reclassification, are initiated, authorities expect laboratories to verify results using a second ELISA method or an orthogonal technique, such as LC–MS/MS. This two‐step confirmation process ensures that regulatory decisions are based on robust and reproducible evidence.

#### 4.2.2 Regulations From Selected Other Countries

Outside Europe, many countries have adopted the Codex‐recommended 20 mg gluten/kg threshold, although national implementations differ in scope and enforcement. Throughout this review, concentrations are expressed as milligrams of gluten per kilogram to maintain consistency with Codex mass fraction terminology. The unit ppm is numerically equivalent for these concentrations, but the text uses mg gluten/kg except where ppm appears in direct quotations or in the wording of national regulations.

In the United States, gluten‐free labeling is regulated by the Food and Drug Administration (FDA) under 21 CFR §101.91 (Electronic Code of Federal Regulations, n.d.). This rule, introduced following the Food Allergen Labeling and Consumer Protection Act of 2004 (FDA 2004), identifies wheat, barley, rye, and their crossbreeds as sources of gluten.

The final rule defines the term “gluten‐free” to mean that “the food bearing the gluten‐free claim does not contain an ingredient that is a gluten‐containing grain (e.g., spelt wheat) or an ingredient that is derived from a gluten‐containing grain and that has not been processed to remove gluten (e.g., wheat flour); or an ingredient that is derived from a gluten‐containing grain and that has been processed to remove gluten (e.g., wheat starch), if the use of that ingredient results in the presence of 20 parts per million (ppm, mg/kg) or more gluten in the food; orinherently does not contain gluten; and that any unavoidable presence of gluten in the food is below 20 ppm gluten.” Since 2020, the regulation has also covered fermented and hydrolyzed foods, for which manufacturers must demonstrate gluten control through validated production processes when direct analytical quantification is not feasible.

In Canada, gluten‐free labeling is regulated under Section B.24.018 of the Food and Drug Regulations (CFIA 2012). This provision prohibits gluten‐free claims on any food that contains any gluten protein or modified gluten protein, including any gluten protein fraction, from wheat, oats, barley, rye, triticale, or their hybridized strains. Although no numerical limit is specified, Health Canada interprets compliance using an operational limit below 20 mg gluten/kg, in alignment with Codex. Oats are permitted to bear the gluten‐free claim when sourced from certified programs that control cross‐contact and maintain gluten content within acceptable limits.

Brazil follows a presence–absence model rather than a quantitative one. Law No. 10.674 (2003) (Presidência da República, 2003) requires all packaged foods to declare gluten status using either contém glúten (contains gluten) or não contém glúten (does not contain gluten). The law does not specify a threshold or consider partial gluten removal, making it a binary system based solely on ingredient composition.

Some countries in Latin America apply stricter standards. Argentina allows the label “libre de gluten” (free from gluten) only if gluten content does not exceed 10 mg gluten/kg and mandates the use of an official national gluten‐free symbol. Chile enforces an even lower limit of 3 mg gluten/kg, combined with mandatory front‐of‐pack labeling and retail‐display controls to minimize cross‐contact. These thresholds represent a more conservative approach to consumer protection but also pose analytical challenges, as few validated methods can reliably quantify gluten at such low concentrations.

In Australia and New Zealand, the regulatory model differs fundamentally from the Codex approach. Under the Food Standards Code, a food labeled “gluten‐free” must contain no detectable gluten, rather than comply with a defined numerical threshold. Compliance, therefore, depends directly on the LOD of the analytical method applied. In practice, this creates a moving regulatory boundary, as improvements in analytical sensitivity may change what is considered compliant. This model places particular emphasis on validated detection capability and method performance characteristics.

Because thresholds and enforcement practices differ internationally, harmonized analytical performance criteria are essential to ensure that test results are comparable across jurisdictions. In parallel with public regulation, private‐sector frameworks have emerged to standardize gluten‐free manufacturing practices within global supply chains. These voluntary standards specify risk assessment procedures, ingredient sourcing, periodic testing, and verification of production environments. They complement public law by providing practical tools for maintaining gluten‐free status in diverse markets. When national limits fall below the quantification capability of current analytical methods—as in Chile's 3 mg gluten/kg or Argentina's 10 mg gluten/kg standards—compliance becomes analytically uncertain. Extraction efficiency, matrix effects, and reproducibility must therefore be evaluated when interpreting results against such ultralow thresholds. Without harmonized performance benchmarks, achieving consistency in enforcement and mutual recognition across jurisdictions remains difficult.

In parallel, Codex is currently considering a 4‐mg reference dose for gluten in wheat as the basis for precautionary allergen labeling (PAL) statements (FAO and WHO 2025b). If adopted, PAL thresholds would constitute a further driver for sensitive and validated quantitative gluten analysis, as compliance would require reliable detection at concentrations substantially below the current 20 mg gluten/kg labeling threshold. This development underlines the urgency of establishing internationally harmonized MPCs applicable across the range of regulatory and precautionary gluten concentrations.

#### 4.2.3 BRCGS Global Standard Gluten‐Free Issue 4

The BRCGS Global Standard Gluten‐Free Issue 4 (BRCGS 2024) builds on ISO/IEC 17021 (ISO, n.d.) and establishes a comprehensive management framework to prevent gluten contamination throughout food production. It sets clear requirements for raw material approval, process controls, traceability, and analytical verification, with strong emphasis on supplier management. Ingredients used in approved gluten‐free products must come from suppliers that can demonstrate effective gluten control measures.

The fourth edition was developed through five Working Group meetings held in 2023, involving representatives from retail, manufacturing, analytical laboratories, coeliac associations, certification bodies, and cereal chemistry experts. This broad collaboration ensured that the Standard reflects both scientific evidence and practical implementation experience.

Analytical testing forms part of a wider risk‐based system. Each certified site must implement a documented sampling and testing plan to verify that gluten cross‐contact is effectively controlled. At a minimum, a laboratory accredited to ISO/IEC 17025 must perform one verification analysis per year. The focus lies on process validation and verification rather than extensive end product testing, although analytical confirmation remains necessary for regulatory assurance and consumer confidence.

##### 4.2.3.1 Audit and Certification

Certification audits against the Standard are conducted by independent third‐party organizations registered with BRCGS and accredited to ISO/IEC 17021. Each certification body must complete a registration process that includes a review of its technical competence and administrative systems. Auditors must meet specific qualification and experience criteria and be formally affiliated with an approved body. Sites seeking certification are expected to either hold an existing certification recognized by the Global Food Safety Initiative (GFSI) or be working toward one at an intermediate level. The GFSI plays a critical role in harmonizing and benchmarking food safety standards by uniting diverse stakeholders from the food sector. Through this collaborative network, GFSI seeks to ensure the integrity, consistency, and global applicability of food safety practices. Certification audits may be conducted as part of a broader GFSI audit, typically extending it by approximately half a day, or as standalone inspections lasting 1–1.5 days. Any nonconformities must be addressed and resolved within 28 calendar days. Following the resolution, the certification report and official status are issued within a 42‐day window from the commencement of the audit.

##### 4.2.3.2 Endorsement by Coeliac Associations

The Standard carries formal endorsements from several coeliac advocacy organizations, namely Celiac Canada, Beyond Celiac (United States), Acelmex (Mexico), and the Association of European Coeliac Societies (AOECS). The AOECS includes 39 national member associations across Europe and seven affiliate members spanning Europe, North America, and the Middle East. These endorsements contribute to the Standard's acceptance by regulators and consumer groups in both Europe and North America. The current version was issued in 2016 and has not been revised since. Its analytical provisions specify the R5 Méndez ELISA as the reference method for gluten determination, consistent with the Codex approach.

##### 4.2.3.3 Comparison With Other Private Gluten‐Free Programs

Unlike more general food safety schemes such as FSSC 22000, SQF, or IFS Food, where gluten is treated under broader allergen management protocols, BRCGS offers the only dedicated gluten‐free certification integrated within the GFSI framework (GFSI, n.d.). The program certifies the integrity of a manufacturer's entire gluten control system, rather than certifying individual products. This system‐level approach aligns well with supply chain needs and with regulatory expectations for preventive controls rather than relying solely on finished‐product guarantees.

##### 4.2.3.4 Relationship to MPCs

Private certification frameworks such as BRCGS operate alongside analytical performance criteria. MPCs define the required levels of accuracy, repeatability, and recovery for gluten test methods. At the same time, the BRCGS Standard ensures that certified sites have documented systems for appropriately applying validated methods within their gluten control programs. This means private standards and scientific performance criteria complement each other. Together, they enable both technical robustness and practical compliance across complex international supply networks. Public regulation and private standards, when properly aligned with validated methods, form a unified framework for gluten‐free assurance. Their continued evolution depends on scientifically sound, internationally harmonized criteria for analytical performance.

## 5 Method Standardization and Performance Criteria

### 5.1 Rationale for MPCs

To enforce gluten‐free regulations effectively, authorities need analytical methods that deliver accurate, reproducible, and comparable results regardless of the laboratory, equipment, or food matrix. Designating a single reference method alone cannot achieve this kind of consistency. This limitation is relevant for gluten because international frameworks have historically relied on a single defining immunoassay approach, which supports harmonization but also introduces method dependency. MPCs provide a way to specify performance expectations that remain applicable when laboratories use different antibodies, extraction procedures, calibration systems, or orthogonal methods in complex and processed matrices. Instead, it requires clearly defined criteria that set measurable expectations for how well any given method must perform. MPCs outline the minimum analytical standards that methods must meet to be deemed fit for regulatory purposes. This includes targets for recovery, repeatability, reproducibility, and detection and quantification limits. Crucially, MPCs do not dictate how an analysis should be performed; they define what performance outcomes must be achieved. This permits the use of various techniques and encourages innovation, provided each method can demonstrate that it meets the required standards.

The Codex Alimentarius Commission formally recognizes the use of performance‐based validation. In its Procedural Manual (FAO and WHO 2025a), Codex recommends that regulatory analytical methods be governed by quantitative performance metrics rather than mandating specific procedures. This principle supports flexibility across technologies while maintaining comparability, reducing the need for regulatory amendments when new methods emerge. Recent FAO/WHO expert consultations on gluten also emphasized the need for such criteria for gluten detection and quantification methods (FAO and WHO 2025b). The panel noted that current analytical methods differ in extraction efficiency, antibody specificity, and matrix‐dependent behavior. It concluded that the development of MPCs is required to ensure reliable quantification across food categories. The consultation further highlighted the importance of expanding access to well‐characterized reference materials and improving transparency regarding assay‐specific reagents, as these factors directly influence measurement uncertainty at levels relevant to risk assessment and regulatory decisions.

### 5.2 Role of CODEX and CCMAS

Responsibility for developing and guiding the application of MPCs lies with the Codex Committee on Methods of Analysis and Sampling (CCMAS). This committee issues international recommendations on method validation, comparison, and selection within Codex standards. Its work underpins national and regional analytical policies worldwide. Codex has already applied MPCs in several high‐impact areas where analytical precision and interlaboratory consistency are essential. For instance, contaminants such as lead, cadmium, and various mycotoxins are regulated under performance‐based criteria laid out in Codex CAC/GL 90–2017 (Codex Alimentarius 2017a). These criteria establish acceptable parameters for repeatability, recovery, and reproducibility. In the domain of pesticide residues, Codex Guidelines (CAC/GL 40–1993, revised 2003) (Codex Alimentarius 1993) set comprehensive requirements for method scope, linearity, and LOQs. Similarly, for microbiological testing, the Codex Guidelines for Validation of Analytical Methods for Food Microorganisms (CAC/GL 74–2010) (Codex Alimentarius 2010) define quantitative parameters for sensitivity, specificity, and reproducibility to ensure consistent detection performance.

These examples demonstrate that performance‐based standardization is well established within Codex methodology. Despite the comparable need for analytical reliability, internationally agreed performance requirements for gluten remain limited and method specific. EN 17254:2019 defines minimum performance requirements for gluten determination by ELISA, but it does not provide method‐independent criteria applicable across different analytical principles (European Standards 2019; Koerner et al. 2013). The absence of such benchmarks means that even validated ELISA methods can yield nonequivalent results, particularly across food types or levels of processing. Establishing Codex‐endorsed MPCs for gluten would extend the same structured, performance‐based framework already applied to contaminants, residues, and microbiological methods, ensuring greater consistency and international alignment.

### 5.3 Statistical Elements of MPCs

MPCs are built around key statistical parameters that define the expected analytical performance under controlled laboratory conditions representative of routine testing. These parameters typically include (Codex Alimentarius 1999):

- Repeatability (r): Agreement between independent results obtained under identical conditions, including the same operator, equipment, and laboratory.
- Reproducibility (R): Agreement between test results generated under varying conditions, such as different laboratories, instruments, or analysts.
- Recovery (%): The proportion of gluten recovered during analysis, expressed as a percentage of the known or nominal concentration in the sample.
- LOD and LOQ: The lowest gluten concentrations that can be reliably detected and quantified, respectively.
- Trueness (bias): The degree to which the mean test result corresponds to a recognized reference value.

Acceptable performance targets for these parameters are established through collaborative validation studies and are generally expressed as statistical ranges. Tools such as the Horwitz equation and the HorRat ratio are commonly used to assess reproducibility, with HorRat values between 0.5 and 2.0 typically considered acceptable for regulatory testing. For gluten quantification, MPCs could specify maximum allowable relative standard deviations for repeatability and reproducibility near the 20 mg gluten/kg regulatory threshold. They could also define acceptable recovery ranges, for example, between 80% and 120%, using incurred reference materials that mimic real processing conditions (AOAC International 2016; Codex Alimentarius 2017b). In practice, PT results show that recovery often falls outside this interval, particularly in processed or complex matrices. Data from the DLA allergen PT scheme indicate recoveries well above 100% in low‐interference matrices, whereas thermally processed and fat‐rich foods show marked reductions relative to spiking levels. FAPAS gluten PT rounds also report substantial between‐kit differences in assigned values within the same PT item, which indicates method‐dependent bias rather than purely random error. These recovery and method effects have direct implications near the 20 mg gluten/kg threshold, because moderate bias can shift a measured value across the decision limit, leading to incorrect classification of products marketed as gluten‐free. This supports the need to consider measurement uncertainty and to confirm results near the threshold using a second ELISA or an orthogonal method, where feasible. These parameters help laboratories and competent authorities interpret results near the threshold consistently.

### 5.4 Technology Neutrality and Analytical Flexibility

Ideally, the MPC framework is designed to be technology neutral. Instead of prescribing a specific analytical format, it defines the required performance outcomes, irrespective of whether a method is based on ELISA, LC–MS/MS, or another platform. This structure promotes technological innovation while maintaining scientific comparability. Although most current gluten analysis relies on immunoassays, emerging technologies such as targeted LC–MS/MS are demonstrating strong potential. Mass spectrometry can achieve sensitivity comparable to ELISA (Xu et al. 2025), with detection limits around 10 mg gluten/kg and the ability to report results as gluten protein equivalents. Provided these methods meet the same MPC‐defined performance benchmarks, they can serve as valid alternatives or orthogonal tools to antibody‐based assays. This allows for more reliable detection in complex matrices, including hydrolyzed and thermally processed foods.

### 5.5 Implementation in Laboratory Practice

Effective implementation of MPCs requires coordination among method developers, analytical laboratories, accreditation bodies, and PT providers. Laboratories accredited under ISO/IEC 17025 already operate within a framework that mandates validation, quality control, and traceable documentation. Integrating MPCs into this structure ensures consistent, verifiable analytical performance. PT schemes operated by FAPAS and DLA under ISO/IEC 17043 provide the comparative datasets necessary to assess whether laboratories meet defined performance requirements. These programs supply incurred, matrix‐matched test materials that replicate the complexity of real food products. When MPCs are used in conjunction with PT, laboratory results can be assessed against predetermined performance limits for recovery, precision, or reproducibility. This provides a transparent and objective measure of analytical capability across different methods and participants.

At the European level, coordination of allergen and gluten testing practices is further supported by the European Network of Food Allergen Detection Laboratories (ENFADL). Established in 2017 in response to calls for greater harmonization of allergen quantification across EU member states, ENFADL brings together national experts, the European Commission's Joint Research Centre (JRC), and representatives from official laboratories. The network prioritizes the development of best practices for analytical methods, reporting, and interlaboratory comparability. Annual meetings facilitated by the JRC provide a platform for exchanging technical experience and for organizing collaborative proficiency tests, thereby strengthening consistency across national laboratory systems.

To promote regulatory alignment, existing validation frameworks, such as ISO/IEC 17025, ISO 13528, and the AOAC INTERNATIONAL Guidelines for Validation of Quantitative Gluten Methods, already incorporate elements consistent with the MPCs. However, formal integration and harmonization of these criteria across systems would strengthen comparability and ensure consistent application in both regulatory and private‐sector testing. Multilaboratory validation across diverse food categories will generate the empirical basis for defining these criteria. Once established, the parameters could be reviewed by the Codex Committee on Methods of Analysis and Sampling (CCMAS) and, if adopted, incorporated into Codex standards to ensure global applicability. By defining common performance criteria for gluten methods, MPCs enable regulators, accreditation bodies, and private certification systems to recognize analytical equivalence across technologies and jurisdictions. This harmonization reduces redundant validation work, supports consistent regulatory decisions, and strengthens transparency in market enforcement.

### 5.6 Advantages of a Performance‐Based Approach

Adopting a performance‐based framework for gluten analysis provides several operational and strategic advantages.

First, it delivers stability and scalability. Once MPCs are established, any analytical method, regardless of technology, can be accepted for regulatory use if it meets the required analytical benchmarks (e.g., precision, recovery, accuracy). This reduces dependence on individual proprietary kits and supports continued innovation by test developers.

Second, it improves data comparability across laboratories and jurisdictions. When all parties assess results against shared performance targets, numerical differences can be interpreted as analytical variation rather than methodological design.

Third, MPCs strengthen transparency and accountability in both public and private testing. Defined performance expectations can be verified during audits, certification, or regulatory inspection. This is particularly relevant for private assurance programs such as BRCGS, which rely on objective evidence of effective gluten control systems. When incorporated into such frameworks, MPCs help ensure that analytical data produced in manufacturing environments meet the same reliability standards as those used by regulators.

Performance‐based criteria enable the integration of advanced methods into gluten analysis without compromising analytical rigor. The performance‐based model supports methodological innovation, regulatory cohesion, and scientific credibility. As food production systems and analytical tools evolve, this framework ensures that gluten detection remains robust, reproducible, and fit for purpose.

### 5.7 Developing MPCs for Gluten Analysis

Developing robust MPCs for gluten will require coordinated international work using agreed validation designs and well‐characterized materials. The recent (November 2025) FAO/WHO expert consultations on gluten emphasized the need for such criteria. They highlighted that variability in extraction efficiency, antibody specificity, and matrix‐dependent recovery must be addressed to ensure reliable quantification across food categories.

A practical pathway would begin with multilaboratory collaborative studies using incurred reference materials that span key food matrices and processing conditions. These studies should generate datasets that allow statistical definition of acceptable ranges for recovery, repeatability, and reproducibility at concentrations close to the 20 mg gluten/kg regulatory threshold. Consistent study design, including harmonized extraction procedures, calibration strategies, and reporting formats, is essential to ensure that results from different laboratories can be combined for criterion setting.

Once performance ranges are derived, a draft set of MPCs can be compiled in accordance with Codex requirements for method performance documentation. These materials could then be submitted to the Codex Committee on Methods of Analysis and Sampling (CCMAS) for consideration. If endorsed, the MPCs could be incorporated into Codex guidance or referenced within CXS 118–1979, providing an internationally recognized benchmark for gluten analysis. This process would allow established and emerging analytical technologies to be evaluated against common performance expectations, while keeping the framework adaptable to future methodological developments.

In parallel, the ILSI Europe Food Allergen Analysis Task Force (ILSI Europe 2025) has initiated work to develop harmonized guidance on the performance of allergen and gluten methods, which can support future Codex‐facing efforts by providing a structured framework for collaborative validation studies.

### 5.8 The Link With Regulations and Private Standards

MPCs provide the quantitative link between public regulation and private gluten control programs, supporting compliance not only with gluten‐free labeling requirements but also with PAL thresholds, where applicable. Public authorities depend on validated methods to verify compliance with labeling laws. At the same time, private schemes such as the BRCGS Global Standard Gluten‐Free rely on analytical verification to confirm process control. Integrating both domains under a common performance‐based framework would enhance consistency across enforcement, certification, and market assurance. MPCs would provide the measurable criteria against which gluten‐testing methods are evaluated, whether for official controls, internal monitoring, or third‐party audits.

For example, a manufacturer certified under BRCGS could verify that its contract laboratory employs an ELISA or LC–MS/MS method meeting Codex‐aligned MPCs for recovery, repeatability, and detection limits. The same criteria could guide regulatory laboratories assessing compliance samples, ensuring comparable expectations across jurisdictions. Such alignment would reduce ambiguity about acceptable test outcomes and promote mutual recognition of analytical data worldwide.

Harmonizing validation expectations across regulatory and private systems would also lessen the compliance burden for laboratories, method developers, and manufacturers. Instead of meeting separate or conflicting requirements, all parties could refer to a single, scientifically defined benchmark applicable across testing contexts—encompassing both gluten‐free labeling thresholds and, should they be adopted, PAL reference doses for gluten.

### 5.9 Outlook

The establishment of MPCs for gluten analysis would align this analytical domain with other areas of food regulation, including contaminants, pesticide residues, and microbiological testing, where performance‐based validation is already standard practice. It would provide a transparent, statistically defined framework that ensures the reliability of analytical results while remaining adaptable to new technologies and product types. Such a framework would reinforce regulatory enforcement by ensuring that laboratories worldwide operate to the same performance expectations. It would also encourage innovation by admitting new analytical technologies once they demonstrate equivalent or superior performance. Most importantly, it would enhance confidence in gluten‐free claims across the entire supply chain, from ingredient sourcing to retail labeling. Combining validated reference materials, statistically defined performance thresholds, and harmonized public and private standards would establish a coherent, internationally recognized system for gluten control. This would close existing gaps in comparability, enforcement, and mutual recognition, supporting both consumer protection and equitable market access.

## 6 Conclusions

The review shows that reliable gluten testing depends on analytical systems that are aligned in their validation requirements, statistical criteria, and reference materials. Differences in extraction efficiency, antibody behavior, matrix effects, and availability of fit‐for‐purpose reference materials continue to create analytical discrepancies and uncertainty near the 20 mg gluten/kg threshold, even when laboratories follow established protocols. Improving the technical foundations of gluten analysis to guarantee analytical accuracy is therefore a higher priority than adjusting the regulatory limit itself.

Representative incurred reference materials are central to this improvement. Materials that reproduce the structure of real foods allow laboratories to evaluate recovery, precision, and measurement uncertainty under conditions that match routine testing. When reference materials are based on a single gluten fraction (e.g., lacking glutelin fractions) or do not capture processing effects, laboratories cannot assess method performance that reflects the range of products on the market.

MPCs provide a practical way to bring consistency to gluten analysis. MPCs define acceptable performance ranges for accuracy, recovery, reproducibility, and measurement uncertainty, regardless of the analytical technology used. When methods meet these criteria, laboratories can interpret results near the threshold with greater confidence. This reduces the likelihood of unjustified product withdrawals or undetected noncompliance. Accurate measurements produced within an MPC framework protect consumers from inadvertent exposure and reduce the financial and reputational risks that manufacturers face when operating across different regulatory systems.

PT schemes play an essential role within this structure. They show how methods behave across laboratories and matrices, which cannot be established through single‐laboratory validation alone. PT data reveal systematic biases, highlight cases where extraction or antibody responses vary across food types, and confirm whether methods that meet defined performance criteria continue to do so under routine conditions.

Private standards, such as the BRCGS Gluten‐Free Standard, provide additional support by requiring the use of methods that have demonstrated fitness for purpose. Their performance‐based approach aligns with an MPC framework and helps manufacturers manage compliance across jurisdictions with varying accreditation and enforcement structures. For producers trading internationally, this reduces the likelihood of inconsistent results and the associated commercial and reputational consequences.

A forward path will depend on practical cooperation between standardization bodies, regulators, laboratories, and PT providers. Clear expectations for validation, reference materials, and method performance would provide laboratories with a sound basis for interpreting results near regulatory thresholds. This would improve the reliability of routine testing, support enforcement, and provide manufacturers with clearer assurance that their products meet gluten‐free requirements across all markets where they operate.

## Author Contributions

**Bert Popping**: conceptualization, writing – review and editing, supervision. **Daniela Bartsch**: writing – review and editing. **Matthias Besler‐Scharf**: writing – review and editing. **Carmen Diaz‐Amigo**: writing – review and editing. **Rupert Hochegger**: writing – review and editing. **Barry Meikle**: funding acquisition, writing – review and editing. **Mark Sykes**: writing – review and editing.

## Conflicts of Interest

The authors declare no conflicts of interest.
